# Altered Spontaneous Neural Activity in Peripartum Depression: A Resting-State Functional Magnetic Resonance Imaging Study

**DOI:** 10.3389/fpsyg.2020.00656

**Published:** 2020-04-08

**Authors:** Kaili Che, Ning Mao, Yuna Li, Meijie Liu, Heng Ma, Wei Bai, Xiao Xu, Jianjun Dong, Ying Li, Yinghong Shi, Haizhu Xie

**Affiliations:** ^1^Department of Radiology, Yantai Yuhuangding Hospital, Qingdao University, Yantai, China; ^2^Clinical Medical College, Binzhou Medical University, Yantai, China; ^3^Medical Imaging Department, Weifang Medical University, Weifang, China; ^4^Department of Radiology, The First Affiliated Hospital of Zhengzhou University, Zhengzhou, China

**Keywords:** peripartum depression, fractional amplitude of low-frequency fluctuation, regional homogeneity, resting-state functional MRI, dorsolateral prefrontal cortex

## Abstract

Abnormalities related to peripartum depression (PPD) have been detected in several brain regions through tasking-state functional magnetic resonance imaging (fMRI). In this study, we used the two markers of resting-state fMRI (rs-fMRI) to investigate changes in spontaneous neural activity of PPD and their correlation with depression severity. A total of 16 individuals with PPD were compared with 16 age- and education-matched healthy controls (HCs) by using rs-fMRI. Two-sample *t*-test was used to compare the fractional amplitudes of low-frequency fluctuation (fALFF) and regional homogeneity (ReHo) values between groups. Pearson correlation analysis was used to determine the correlation between the fALFF and ReHo of the abnormal brain region and the Hamilton Depression Scale (HAMD) and Edinburgh Postnatal Depression Scale scores. The spontaneous neural activity of the PPD group significantly increased mainly in the left middle frontal gyrus, left precuneus, left inferior parietal lobule, and left dorsolateral prefrontal cortex (DLPFC) and decreased mainly in the bilateral precentral gyrus and right inferior occipital gyrus compared with those of the HCs. The fALFF value of the left DLPFC was negatively correlated with the HAMD score in PPD. This rs-fMRI study suggests that changes in the spontaneous neural activity of these regions are related to emotional responses. PPD cases with low fALFF values in the left DLPFC have severe depression.

## Introduction

The peripartum period is characterized by normal variations in psychological functioning and physiology and is a complicated phase in a woman’s life (Duan et al., 2017). Depression during perinatal and postpartum periods is referred to as peripartum depression (PPD). However, no agreement has been reached on the exact duration of PPD after giving birth (Lee and Chung, 2007). PPD is a major public health problem worldwide (Wisner et al., 2006). Maternal depressive symptoms affect most women, and 7.4–20% of women experience marked depressive symptoms at different periods of pregnancy (Bennett et al., 2004). PPD affects one in eight women (Cox et al., 1987) and causes disability, endangers life, affects the relationship between a mother and her infant, and negatively affects the social, emotional, and cognitive development of future generations (Beck, 1998). Normal peripartum-related physiological changes in the brain structure, function, and metabolism can be understood using non-invasive resting-state functional magnetic resonance imaging (rs-fMRI) (Raichle, 2001). Discovering voxel-based biomarkers from the rs-fMRI of patients with PPD is useful in understanding neurological changes that may contribute to developing new treatment methods.

Tasking-state fMRI was performed to explore the effects of emotional responses on neural activity and functional connectivity during PPD. Clinical depressive symptoms are related to reduced amygdala responsivity with positive stimuli (Barrett et al., 2012) in the postpartum stage, threat-related stimuli (Silverman et al., 2011), and negatively valenced stimuli (Silverman et al., 2007; Moses-Kolko et al., 2010). Several abnormalities in ventral striatal activity after reward (Moses-Kolko et al., 2011), increased amygdala activity in positive emotional stimuli (Wonch et al., 2016), and reduced inferior frontal gyrus with negative stimuli were observed (Bannbers et al., 2013). Mothers with a history of depression have significantly reduced activity in the middle thalamus, and this condition is often accompanied by maternal hemodynamic abnormalities (Laurent and Ablow, 2012). Tasking-state fMRI can find the abnormalities at the functional level and aids in the study of the pathophysiological mechanisms of PPD. However, tasking-state fMRI requires full cooperation of the subjects during scanning. The cognitive ability, comprehension skill and education level of the subjects affect the test results, and the process is relatively complex. The study of the brain network is also limited. rs-fMRI has been used for the disruption of functional connectivity networks in women with postpartum depression. Reduced connectivity between the anterior cingulate cortex and the left dorsolateral prefrontal cortex and bilateral amygdale (Deligiannidis et al., 2013) and reduced posterior cingulate cortex-right amygdala connectivity have been observed in women with postpartum depression (Chase et al., 2014). Within the default mode network (DMN) identified with independent component analysis, a significant group difference in the dorsomedial prefrontal cortex (DMPFC) was identified via dual-regression analysis; this region demonstrated greater connectivity with the rest of the DMN in PPD compared with healthy women (Deligiannidis et al., 2019). PPD exhibited significantly decreased voxel-mirrored homotopic connectivity values in the bilateral DMPFC, dorsal anterior cingulate cortex, and orbitofrontal cortex (Zhang et al., 2020). Functional links are generally used in calculating temporal synchronization between regions of interest and other brain regions and reflecting changes in whole-brain function. However, regions of interest are selected on the basis of different prioris, limiting the validation of different studies.

The fractional amplitude of low frequency fluctuation (fALFF) and regional homogeneity (ReHo) are the major methods for studying local spontaneous neural activity. fALFF is useful in identifying specific local brain areas with abnormal blood oxygen level-dependent signal and activity, whereas ReHo analysis reflects the consistency of brain activity in a time series (Zang et al., 2004). At present, fALFF and ReHo have not been combined for exploring the neurological mechanism of PPD. This study used two methods to reveal the general features of abnormal brain function in humans versus either method alone.

## Materials and Methods

### Subjects

This study was approved by the Research Ethics Committee of the Yantai Yuhuangding Hospital of Shandong Province, China. All subjects signed informed consent.

This study was conducted from September 2017 to July 2019 and recruited 16 individuals with PPD from the psychological clinic of Yantai Yuhuangding Hospital, Qingdao University. The inclusive criteria were as follows: (1) meeting the diagnostic criteria for Diagnostic and Statistical Manual of Mental Disorders, Fifth Edition unipolar depression; (2) first-onset patients without medication; (3) Hamilton Depression Scale (HAMD) score (Hamilton, 1967) >20 points; (4) Edinburgh Postnatal Depression Scale (EPDS) score (Cox et al., 1987) ≥12 points; (5) right-handedness; (6) depression onset during pregnancy and new-onset postpartum (patients newly diagnosed with depression during pregnancy and postpartum were considered to maximize generality) (Stowe et al., 2005); (7) onset of 1 year after childbirth (healthy full-term infants) (Stuart-Parrigon and Stuart, 2014); (8) no MRI examination contraindications and abnormalities in MRI structure imaging. The exclusion criteria were as follows: (1) past and present medical history of psychiatric or neurological disorders in patients and first-degree relatives; (2) any severely unstable disease requiring medical treatment or hospitalization; (3) history of drug abuse or drug dependence within 1 year; (4) hormone contraceptives; (5) left-handedness; (6) history of craniocerebral trauma; (7) complications, such as hypertension, diabetes, eclampsia, heart disease, or postpartum hemorrhage that occurred during pregnancy or childbirth.

Sixteen healthy postpartum women as healthy controls (HCs) that matched the PPD group in terms of age, education level, and body mass index (BMI) were selected from a local community. The inclusion criteria were as follows: (1) no history of depressive episodes; (2) no history of using antidepressants and other antipsychotics; (3) HAMD score < 8 points; (4) EPDS score < 3 points. The exclusion criteria were the same as those in the PPD group.

A total of 32 subjects were assessed for the severity of depression by using HAMD and EPDS before scanning. HAMD is the most widely used scale in the clinical assessment of depression and includes 24 items. EPDS is a self-rating scale with 10 items. The HAMD scale was administered and independently scored by two psychologists. Subjects with HAMD scores > 20 and EPDS scores ≥ 12 were included in the PPD group.

### MRI Data Acquisition

Scanning was performed using a GE MR750W (GE Healthcare, United States) device and an eight-channel receiver array head coil. Fillers and earplugs were used to reduce head movement and scanner noise. The subjects were instructed to close their eyes and rest, avoid thinking about anything, and avoid any head movement during the scan. First, high-resolution 3D T1-weighted structural images were acquired [repetation time (TR) = 8.2 ms, echo time (TE) = 3.2 ms, field of view (FOV) = 256 × 256 mm^2^, slice thickness = 1 mm, matrix = 256 × 256], and T2 phase data were collected. The axial rs-fMRI image was obtained using a gradient echo-planar imaging sequence. The specific parameters were as follows: TR = 2000 ms, TE = 30 ms, slices = 36, slice thickness = 3 mm, gap = 0 mm, flip angle = 90°, FOV = 240 × 240 mm^2^, matrix = 64 × 64. After the MRI scan, all subjects were asked whether they were asleep or distracted during the scan for the exclusion of unqualified subjects.

### Functional Data Preprocessing

All brain function data were analyzed using SPM8 and REST2 software. The first 10 volumes from each time series were discarded to eliminate the effects of inadaptability and magnetic field inhomogeneity. The remaining data were included in the follow-up analysis. Slice timing and realignment were performed on each subject. Head-motion correction was carried out to artificially remove data whose head-motion was >1.0 mm and rotation was >1.0°. The remaining dataset was spatially normalized to the Montreal Neurological Institute template. Each voxel was resampled to 3 mm × 3 mm × 3 mm. The influence of physiological noise was eliminated via linear trend and bandpass filtering (0.01–0.08 Hz) (Liu F. et al., 2012).

### fALFF and ReHo Analyses

Fractional amplitudes of low-frequency fluctuation analysis was performed by smoothing via a Gaussian function with 4 mm full width at half maximum. The time series was first converted to the frequency domain power spectrum by fast Fourier transform. The area under the peak of the power spectrum could be regarded as the energy of the signal. The ALFF of the signal was obtained via root-mean-square calculations on the power spectrum at the range of 0.01–0.08 Hz. The value of ALFF in this range was added to obtain the total ALFF value, and the fALFF value was obtained by dividing the total value of full-band amplitude from 0.01 to 0.25 Hz.

Regional homogeneity was calculated by Kendall’s coefficient concordance (KCC), which reflects the temporal consistency of neural activity in a region of the brain. ReHo maps were normalized by dividing the KCC among each voxel by the global mean ReHo value. The resulting data were spatially smoothed by convolution with a 4 mm full width at half maximum Gaussian kernel.

### Statistical Analysis

Differences between the demographic data of the two groups were analyzed through two-sampled *t*-test performed on SPSS 22.0 (*p* < 0.05 indicates statistical significance). Two-sampled *t*-test was performed to analyze the difference between the two groups of fALFF and ReHo values. Age, BMI and education years of each subject was included as covariates. The covariate regression method was used to eliminate the gray matter volumes, white matter signal, cerebrospinal fluid signal, and whole brain signal. The resulting statistical map was corrected for multiple comparison correction to a significant level of *p* < 0.05. Multiple comparison correction was performed using false discovery rate criterion, and the individual voxel *p* < 0.005 and cluster size >10 voxels were combined. The results were displayed by the ch2.nii template, which is a MRIcron software template, that is, the standard and well-known Colin27 template. The ReHo and fALFF values were extracted from the above differentiated brain regions, and correlation analysis was performed for the HAMD and EPDS scores. The relationship between the values of fALFF and ReHo was studied via Pearson correlation of the two methods. *p* < 0.05 was considered statistically significant.

## Results

### Demographic Characteristics

Table 1 shows the demographic data of the subjects. No significant differences were observed in terms of age (*t* = −0.223, *p* = 0.934), education years (*t* = 0.603, *p* = 0.557), and BMI (*t* = −0.073, *p* = 0.942) between the two groups (*p* > 0.05).

**TABLE 1:** Demographic and depression rating scale of the subjects.

	PPD (*n* = 16)	HCs (*n* = 16)	*P* value
Age (year)	31.16 ± 2.56	31.06 ± 4.42	0.934*
BMI	23.60 ± 3.72	23.70 ± 3.67	0.942*
Education level (years)	15.25 ± 2.82	16.06 ± 2.54	0.557*
HAMD score	31.69 ± 8.61	5.31 ± 2.64	<0.001
EPDS score	16.13 ± 3.34	2.61 ± 0.31	<0.001

Data are expressed as mean ± standard deviation. PPD, peripartum depression; HCs, healthy controls; HAMD, hamilton depression rating scale; EPDS, edinburgh postnatal depression scale; BMI, body mass index. *p < 0.05. All experiments were performed thrice.

### fALFF and ReHo Analyses

**FIGURE 1:**
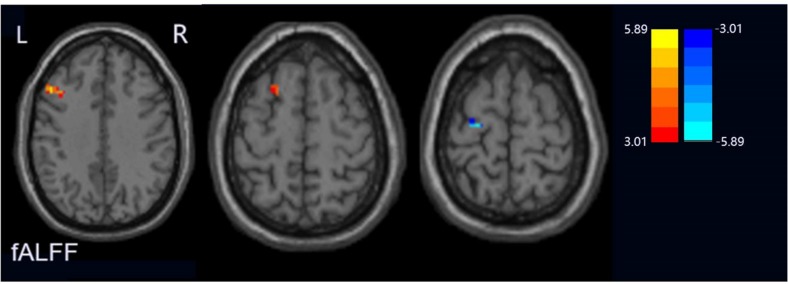
Regions exhibiting differences in fALFF between peripartum depression and healthy postpartum women (p < 0.005 corrected by FDR). The warm colors represented the significance of higher fALEF values and the cool colors represented the significance of lower fALFF values for the group comparisons.

**FIGURE 2:**
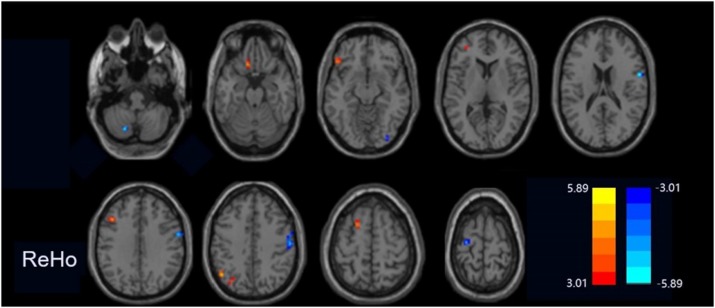
Regions exhibiting differences in ReHo between peripartum depression and healthy postpartum women (p < 0.005 corrected by FDR). The warm colors represented the significance of higher ReHo values and the cool colors represented the significance of lower ReHo values for the group comparisons.

Compared with the HCs group, the PPD group showed increased fALFF values in the left middle frontal gyrus and dorsolateral prefrontal cortex (DLPFC) and decreased fALFF values in the left precentral gyrus (Figure 1). The ReHo values significantly increased in the left cerebrum (orbital part superior frontal gyrus, orbital part inferior frontal gyrus, middle frontal gyrus, precuneus, inferior parietal lobule, and superior frontal gyrus) in the PPD group relative to those of HCs. The ReHo values decreased in the right cerebrum (inferior occipital gyrus and inferior frontal gyrus), bilateral precentral gyrus, and the left cerebellum inferior semilunar lobule (Figure 2). Table 2 shows the different brain regions and specific information between the two groups. The above results were significant (*p* < 0.005).

**TABLE 2:** Regions showing significant differences in fALFF and ReHo values between PPD and HCs.

		MNI peak point coordinates				
Brain regions	BA	X	Y	Z	*t*-value	Voxels
**fALFF differences**						
MFG.L	46	–48	21	36	5.331	31
DLPFC. L	46	–24	15	63	4.282	13
PreCG.L	6	–24	–15	66	–4.530	20
**ReHo differences**						
PL. L	–	–21	–69	–45	–4.690	14
ORBsup. L	11	–12	24	–21	4.495	14
ORBinf. L	38	–45	33	–15	4.347	19
IOG.R	18	33	–87	–9	–4.182	14
MFG. L	46	–33	45	9	4.272	12
IFG. R	44	60	3	18	–4.724	12
P. L	7	–33	–75	39	4.382	22
IPL. L	40	–48	–60	12	4.748	14
PreCG. R	6	60	0	36	–4.868	95
SFG.L	10	–18	–12	57	4.617	14
PreCG. L	6	–27	–15	69	–3.988	14

*The threshold was set at a *p* < 0.005 (FDR corrected). PPD, peripartum depression; HCs, healthy controls. MNI, montreal neurological institute space; BA, Brodmann’s area; -, no-corresponding brain area. MFG.L, left middle frontal gyrus; DLPFC.L, left dorsolateral prefrontal cortex; PreCG.L, left precentral gyrus; PL. L, left cerebellum inferior semi-lunar lobule; ORBsup. L, left superior frontal gyrus, orbital part; ORBinf. L, left inferior frontal gyrus, orbital part; IOG.R, right Inferior occipital gyrus; IFG. R, right inferior frontal gyrus; P. L, left precuneus; IPL. L, left inferior parietal lobule; PreCG. R, right precentral gyrus; SFG.L, left superior frontal gyrus; PreCG. L, left precentral gyrus.*

### Correlation Analysis

**FIGURE 3:**
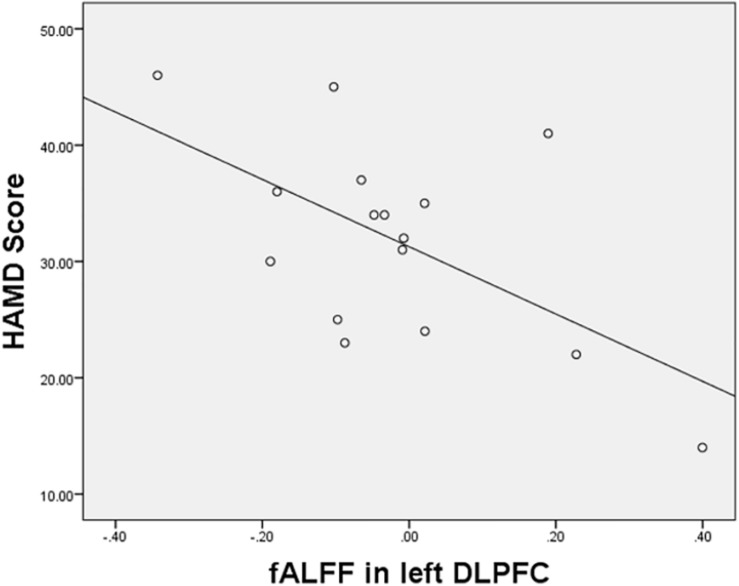
Correlations between the HAMD scores and increased fALFF values in the left DLPFC.

The fALFF values in the DLPFC and the HAMD scores in women with PPD were negatively correlated (*r* = −0.587, *p* = 0.017; Figure 3). No significant correlation was found between other brain regions of the PPD group.

## Discussion

We selected healthy mothers and those with unmedicated PPD by using fALFF and ReHo to explore the changes in the local neural spontaneous activity and the correlation with the degrees of depression in patients with PPD to further understand the PPD neural mechanism. The results showed that patients with PPD had significantly increased spontaneous neural activity in the left cerebrum (DLPFC, orbital part superior frontal gyrus, orbital part of inferior frontal gyrus, middle frontal gyrus, precuneus, inferior parietal lobule, and superior frontal gyrus) relative to that of HCs. Decreased activity was observed in the bilateral precentral gyrus, right inferior occipital gyrus, right inferior frontal gyrus, and left cerebellum inferior semilunar lobule. In addition, the fALFF value of the left DLPFC was negatively related to HAMD scores in the PPD group. These region-specific changes in neural activity may play a key role in maternal depressive symptoms.

Fractional amplitudes of low-frequency fluctuation and ReHo are common analytical methods that have been widely used to investigate the underlying pathogenesis of various neuropsychiatric disorders, particularly depressive disorder (Yao et al., 2009; Liu C.H. et al., 2012; Liu C.H. et al., 2013). In the current research, two methods were used to explore the neurological mechanism of PPD and to discover its precise treatments.

Abnormalities in the local neural activity in several brain regions of DMN, mainly in the left precuneus and left inferior parietal lobule, in the PPD group were detected through ReHo analysis and compared with those in the HCs. DMN is a large-scale brain network comprising a specific set of brain regions, including the ventromedial prefrontal cortex, dorsal medial prefrontal cortex, posterior cingulate cortex/precuneus, ventral anterior cingulate cortex, lateral temporal cortex, and inferior parietal lobule (Raichle et al., 2001; Greicius et al., 2003; Andrews-Hanna et al., 2010; Liu C.H. et al., 2012). In several areas of DMN, the precuneus is a central node responsible for situational memory, consciousness, and awareness (Andrews-Hanna et al., 2014). Morgan et al. (2017) revealed that high levels of positive caregiving are related to increased activation in the precuneus in response to happy faces for mothers with low depressive symptoms. Guo et al. (2013) noted that the functional connection between the left inferior parietal lobule and the cerebellum is weakened, and this condition may be a manifestation of major depressive disorder. The above studies suggest that abnormalities in precuneus may also play a key role in the neural circuits of PPD. The role of the inferior parietal lobule in PPD or major depression disorder needs further study. The correlation between ReHo values and HAMD scores in this part of the brain has not been found in this study. However, this negative finding may be due to the narrow range of depression scores in this trial. Based on the above statement, abnormal ReHo values in the left precuneus may be a characteristic or status biomarker associated with PPD. However, further studies are needed to confirm this hypothesis.

The pathophysiology of major depressive disorder is possibly associated with an abnormal prefrontal cortex (Lacerda et al., 2004). The prefrontal cortex includes the DLPFC, orbitofrontal cortex, medial prefrontal cortex, and anterior cingulated (Fuster, 1999). The prefrontal cortex is mainly responsible for executive and cognitive functions (Yuan and Raz, 2014). In the current experiment, patients with PPD showed increased the left DLPFC fALFF values and increased the ReHo values in the left middle frontal gyrus and superior frontal gyrus. The middle frontal gyrus and superior frontal gyrus constitute the DLPFC, which is one of the most important brain regions of cognitive control and participates in advanced cognitive adjustment, execution, decision making, and other functions (Hansel and von Kanel, 2008). Rosa et al. (2017) suggested the glutamatergic dysfunction and neuronal damage in the DLPFC of patients with PPD similar to other subtypes of depressive disorders, as indicated by magnetic resonance spectroscopy findings. Liston et al. (2014) observed that the superior frontal gyrus is part of the cognitive motor circuit involved in the selection and stimulation of behavioral responses to emotional stimuli, the ease of positive and negative emotion regulation, and is associated with depression. In the current study, the fALFF value of the left DLPFC was negatively correlated with HAMD score, suggesting that the middle frontal gyrus and superior frontal gyrus fALFF or ReHo values may be related to the severity of PPD. Combined with our findings, the local neural activity abnormalities in these regions are closely related to the severity of maternal depression. The orbital prefrontal cortex is a key factor of emotion. Lesions in the orbital prefrontal cortex can lead to depression manifested by emotional instability (irritability, anger, or excitement), impulsivity, multifacetedness, and inappropriate sexual activity. Therefore, “high inhibition” may be related to enhanced orbital activity and control of the limbic system (Biver et al., 1994). Depressive symptoms are associated with the insula and orbital prefrontal cortex when the mother’s response to the happy face of her baby is suppressed (Laurent and Ablow, 2013). The ReHo values in the inferior and superior orbitofrontal cortex increased in PPD, suggesting an increase in neural activity in specific areas, which may be important for understanding the neurobiology of PPD.

The present study discovered decreases in the ReHo values of the bilateral precentral gyrus and inferior frontal gyrus and fALFF values of the left precentral gyrus. Subjective pleasantness is related to the precentral gyrus, right cerebellum, and right inferior frontal gyrus (Kuhn and Gallinat, 2012). A neuroimaging study reported that the precentral gyrus is involved in major depression disorder (Wang et al., 2015). Several studies have confirmed the association between psychomotor retardation, poor action planning, and alterations in the precentral gyrus (Buyukdura et al., 2011). Therefore, the abnormal ReHo or fALFF values of the precentral gyrus reported in the present study may be related to depressive symptoms. The ReHo values of the left cerebellum inferior semilunar lobule decreased in PPD relative to the HCs of our study. The anterior lobe of the cerebellum is mainly related to exercise learning and coordination, whereas the posterior cerebellum is related to human emotion, consciousness, and cognitive processing (Laricchiuta et al., 2015; Olivito et al., 2018). This result differs from that of a study on major depression disorder; Liu F. et al. (2013) noted the decreased fALFF value of the right posterior cerebellar lobe. Thus, the cerebellum may be involved in the neurological mechanism of PPD. However, whether this conjecture is correct remains to be further studied.

The present study has the following limitations. (1) We detected several abnormal brain areas related to PPD and their relationship with clinical features. However, these positive results should be interpreted with caution because they are related to sample size. The sample size of this study is small. We are still collecting cases to expand the sample size to verify the current findings. (2) This cross-sectional study did not involve comparison of fMRI before and after pregnancy. Thus, a direct causal relationship between altered spontaneous neural activity and PPD is impossible to establish. These measures are needed for longitudinal research to analyze and trace the disease before and after its onset. (3) In addition to spontaneous neural activity in the brain, brain function networks should be examined to obtain comprehensive information about mothers with postpartum depression.

## Conclusion

This study explored the changes in brain function and its correlation with depression degree in patients with PPD via fALFF and ReHo analyses. This study provides a new perspective for exploring brain abnormalities in PPD and contributes to the understanding of its neurobiology.

## Data Availability Statement

All datasets generated for this study are included in the article/supplementary material.

## Ethics Statement

The studies involving human participants were reviewed and approved by the Ethics Committee of the Yantai Yuhuangding Hospital. The patients/participants provided their written informed consent to participate in this study approval no. 298:[2019].

## Author Contributions

KC and NM designed the experiment, collected the data, performed the analyses, and wrote the manuscript. YL, ML, HM, WB, XX, YL, and JD collected the data. YS and HX contributed to the discussion and manuscript revision.

## Conflict of Interest

The authors declare that the research was conducted in the absence of any commercial or financial relationships that could be construed as a potential conflict of interest.
